# Nanoscale Bacteria‐Enabled Autonomous Drug Delivery System (NanoBEADS) Enhances Intratumoral Transport of Nanomedicine

**DOI:** 10.1002/advs.201801309

**Published:** 2018-12-05

**Authors:** SeungBeum Suh, Ami Jo, Mahama A. Traore, Ying Zhan, Sheryl L. Coutermarsh‐Ott, Veronica M. Ringel‐Scaia, Irving C. Allen, Richey M. Davis, Bahareh Behkam

**Affiliations:** ^1^ Department of Mechanical Engineering Virginia Tech Blacksburg VA 24061 USA; ^2^ Department of Chemical Engineering Macromolecules Innovation Institute Virginia Tech Blacksburg VA 24061 USA; ^3^ Department of Biomedical Sciences and Pathobiology Virginia Tech Blacksburg VA 24061 USA; ^4^ Macromolecules Innovation Institute School of Biomedical Engineering & Sciences Virginia Tech Blacksburg VA 24061 USA

**Keywords:** bacteria‐based therapies, biohybrid systems, extravascular transport, intratumoral penetration, *Salmonella enterica* serovar Typhimurium, tumor‐targeting bacteria

## Abstract

Cancer drug delivery remains a formidable challenge due to systemic toxicity and inadequate extravascular transport of nanotherapeutics to cells distal from blood vessels. It is hypothesized that, in absence of an external driving force, the *Salmonella enterica* serovar Typhimurium could be exploited for autonomous targeted delivery of nanotherapeutics to currently unreachable sites. To test the hypothesis, a nanoscale bacteria‐enabled autonomous drug delivery system (NanoBEADS) is developed in which the functional capabilities of the tumor‐targeting *S*. Typhimurium VNP20009 are interfaced with poly(lactic‐co‐glycolic acid) nanoparticles. The impact of nanoparticle conjugation is evaluated on NanoBEADS' invasion of cancer cells and intratumoral transport in 3D tumor spheroids in vitro, and biodistribution in a mammary tumor model in vivo. It is found that intercellular (between cells) self‐replication and translocation are the dominant mechanisms of bacteria intratumoral penetration and that nanoparticle conjugation does not impede bacteria's intratumoral transport performance. Through the development of new transport metrics, it is demonstrated that NanoBEADS enhance nanoparticle retention and distribution in solid tumors by up to a remarkable 100‐fold without requiring any externally applied driving force or control input. Such autonomous biohybrid systems could unlock a powerful new paradigm in cancer treatment by improving the therapeutic index of chemotherapeutic drugs and minimizing systemic side effects.

## 1 Introduction

Nanoparticle delivery systems have the potential to significantly enhance cancer therapy through improved targeting, reduced systemic toxicity, regulation of therapeutic residence time in circulation, and preservation of therapeutics' bioavailability. Optimization of nanotherapeutics with respect to vesicle size, geometry, binding, cellular uptake, degradation, and release rates continues to enhance their efficacy.^1^ However, several physical and biological barriers limit delivery of systemically administered nanoparticles to tumors as well as extravascular transport of nanotherapeutics within the tumor, thereby impeding therapy.^2^, ^3^, ^4^, ^5^, ^6^, ^7^ In particular, highly irregular structure and function of the tumor microvasculature coupled with impaired lymphatic drainage elevates interstitial fluid pressure to levels comparable with intravascular pressure and diminishes the interstitial pressure gradient.^2^, ^5^ This combined with a densely structured extracellular matrix, an unusually high fraction of stromal cells, accumulated compressive tissue stress, and expanded intercapillary spaces impose formidable barriers to convective transport of macromolecular chemotherapeutic drugs within the tumor interstitium, precluding therapeutic delivery to cells distal from functioning blood vessels.^4^, ^8^, ^9^, ^10^ To eradicate tumors, therapeutic agents must disperse throughout the cancerous tissue in sufficiently high concentrations and eliminate every malignant cell. Overcoming the aforementioned transport barriers would significantly improve the efficacy of nanomedicine for cancer therapy.

**Figure 1:**
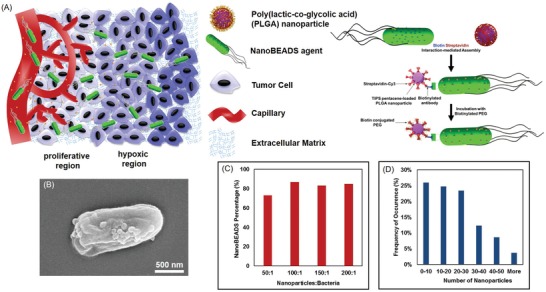
Nanoscale bacteria‐enabled autonomous drug delivery system (NanoBEADS). A) Schematic illustrating enhanced penetration of NanoBEADS in poorly vascularized tumor tissue compared with the passively diffusing nanoparticles (left). Each NanoBEADS agent is constructed by conjugating several streptavidin‐coated PLGA nanoparticles with a tumor targeting biotinylated‐antibody coated *S*. Typhimurium VNP20009, using streptavidin–biotin noncovalent affinity‐based bonds. NanoBEADS assembly was followed by incubation with mPEG‐biotin to quench residual streptavidin binding sites on the nanoparticles. B) A representative SEM image of a NanoBEADS agent. C) Percentage occurrence of NanoBEADS formation at various nanoparticle:bacteria ratios used for NanoBEADS construction. D) Distribution of nanoparticle loading of NanoBEADS agents constructed at nanoparticle to bacteria ratio of 100:1 (*n* = 80).

*Salmonella enterica* serovar Typhimurium VNP20009,^11^, ^12^ an attenuated auxotrophic mutant of the facultative anaerobe *S*. Typhimurium 14028, has been demonstrated to have efficient targeting of solid tumors and metastatic cancer, a good safety profile, and effective tumor penetration mechanisms,^13^, ^14^, ^15^ although complete tumor regression through bacteria monotherapy has not been yet achieved (for a complete review of bacteria‐based cancer therapy, refer to refs. 16, 17, 18). We hypothesized that in the absence of a directed migration mechanism (e.g., chemotaxis^19^ or magnetotaxis^20^), bacteria translocation and proliferation could be exploited to autonomously deliver nanotherapeutics to poorly vascularized and hypoxic tumor tissue, currently inaccessible to passively diffusing nanotherapeutics. To test our hypothesis, we utilized our previously developed bioconjugation method based on streptavidin–biotin interaction^21^, ^22^, ^23^ to bind an ensemble of poly(lactic‐co‐glycolic acid) (PLGA) nanoparticles to the outer membrane of a live tumor‐targeting *S*. Typhimurium VNP20009 bacterium and constructed a nanoscale bacteria‐enabled autonomous drug delivery system (NanoBEADS) agent, as shown in **Figure** **1**A. We first characterized the nanoparticle loading capacity, viability, and growth rate of the NanoBEADS agents. We then evaluated the impact of nanoparticle conjugation on the cancer cell invasion and intratumoral transport of NanoBEADS agents in various 3D tumor spheroid models in vitro, and biodistribution in a mammary breast cancer model in vivo.

We found that intercellular (i.e., between cells) translocation is the dominant mode of bacteria intratumoral penetration and that nanoparticle conjugation does not impede bacteria intratumoral transport performance. Through the development of a set of quantitative metrics for the spatial distribution of therapeutics within the tissue, we demonstrate that bacteria can effectively overcome the barriers to extravascular transport and enhance nanoparticles retention and distribution in solid tumors by up to a remarkable 100‐fold, compared to their passively diffusing counterparts.

The NanoBEADS platform provides a uniquely advantageous approach to the bacteria‐mediated delivery of therapeutic and diagnostic agents. NanoBEADS construction does not require genetic engineering of the bacteria for different cargo. Furthermore, NanoBEADS agents do not rely on any external actuation or control mechanisms and can autonomously transport and distribute nanoparticles within the tumor tissue. We envision that such biohybrid systems could unlock a powerful new paradigm in cancer treatment through specific targeting and efficient delivery of nanotherapeutics into sites difficult to access from circulation, thus improving the therapeutic index of chemotherapeutic drugs with unfavorable pharmacokinetics and minimizing systemic side effects by reducing the required dosage.

## 2 Results and Discussion

### 2.1 Preparation of PLGA Nanoparticles

Biodegradable PLGA nanoparticles were prepared using a nanoprecipitation method.^24^ An organic solution of PLGA and fluorophore 6,13‐bis(triisopropylsylylethynyl) pentacene (TIPS pentacene) was added dropwise into an aqueous solution of Pluronic F127 [(PEG)_97_‐(PPG)_56_‐(PEG)_97_] to form core–shell structures made up by a PLGA core encapsulating the TIPS pentacene dye and a polyethylene glycol (PEG) brush stabilizing the surface, as the polypropylene glycol (PPG) physisorbs to the PLGA by hydrophobic interactions. The nanoparticle diameter was controlled to ≈120 nm with a zeta potential of −26 ± 5 mV. Nanoparticles were monomodal (polydispersity index: 0.11) and had a spherical morphology, as determined by dynamic light scattering (DLS, Figure S1, Supporting Information) and scanning electron microscopy (SEM, Figure 1B), respectively. TIPS pentacene was encapsulated at 0.9 wt% with respect to the PLGA in the nanoparticles to facilitate fluorescence imaging. This fluorophore encapsulated in the PLGA nanoparticles did not affect the viability of the bacteria and cancer cells used in this study (Figures S2 and S3, Supporting Information).

### 2.2 Biomanufacturing of NanoBEADS

Each NanoBEADS agent is comprised of a tumor‐targeting *S*. Typhimurium VNP20009 bacterium conjugated with an ensemble of PLGA nanoparticles. The facultative anaerobe *S*. Typhimurium VNP20009^25^ was selected due to: i) its ability to grow within both the viable and the hypoxic areas of tumors, ii) its genetically stable attenuated virulence, reduction of septic shock potential, and antibiotic susceptibility, achieved by deletion of the *msbB* gene to modify lipid A synthesis,^15^ iii) its purine auxotrophy, conferred by *purI* deletion, that is met within the tumor environment causing preferential bacterial colonization in tumor as compared with normal tissue and rapid clearance from circulation and other organ sites,^11^ iv) its broad application potential in cancer therapy,^26^ and v) its good safety profile in phase I human trials with maximum tolerated dosage of 3 × 10^8^ CFU m^−2^.^13^, ^14^ We adapted the previously developed^21^, ^22^, ^23^, ^27^ bioconjugation method of streptavidin–biotin noncovalent affinity‐based bonds to conjugate streptavidin‐coated PLGA nanoparticles to *S*. Typhimurium VNP20009 coated with biotinylated‐antibody and to construct NanoBEADS (Figure 1A,B, see the Experimental Section). Subsequently, NanoBEADS were incubated with mPEG‐biotin to passivate residual streptavidin binding sites on the nanoparticles. The PEG coating also reduces nonspecific interactions with biological tissues and fluids by increasing the steric distance.^28^ We first investigated the role of antibody concentration on the NanoBEADS formation outcome using SEM imaging (Figure 1B, see the Experimental Section). The fraction of bacteria with at least one nanoparticle attached steadily rose with increasing concentration of antibody up to the maximum antibody concentration of 10 µg mL^−1^ (Figure S4, Supporting Information). We next investigated the effect of the nanoparticle to bacteria ratio during the assembly process. The probability of NanoBEADS formation increased with the increase of the nanoparticle to bacteria ratio up to a ratio of 100:1, beyond which no appreciable change was observed (Figure 1C). Hence, all the NanoBEADS agents were prepared at 10 µg mL^−1^ antibody concentration and 100:1 nanoparticle to bacteria ratio.

### 2.3 Characterization of NanoBEADS

Quantifying the particle loading capacity of the NanoBEADS is important for estimating the drug delivery dosage and determining the overall system efficacy. We quantitated the number of conjugated nanoparticles on each bacterium using SEM imaging (Figure 1B). The distribution of nanoparticle loading is shown in Figure 1D. The estimated number of particles attached to bacteria for a single NanoBEADS agent was 22 ± 14 (*n* = 80). Nanoparticle conjugation did not impact the viability of the bacteria (Figure S2, Supporting Information). Next, we assessed the effect of the bacterial surface modification on its growth. The growth rates of unmodified VNP20009, PEGylated bacteria (see Experimental Section for preparation detail), NanoBEADS under ideal microbiological culture conditions were determined through serial dilution and colony counting. Surface modification increased bacteria doubling time from 43 min for unmodified bacteria to 58 min for PEGylated bacteria and 121 min for the NanoBEADS (Figure S5A, Supporting Information). The slower growth of surface modified bacteria is consistent with previous reports on the effect of nanoparticle conjugation on bacteria growth.^29^ We then evaluated the temporal changes in nanoparticle loading of NanoBEADS agents during proliferation using SEM imaging. We observed that upon binary fission, the nanoparticles remain stably bound to the cell membrane and divide amongst daughter cells (Figure S5B, Supporting Information).

### 2.4 Intratumoral Transport of Therapeutic Agents in 3D Multicellular Tumor Spheroids

We next wanted to test our hypothesis that in the absence of a directed migration mechanism (e.g., chemotaxis^19^ or magnetotaxis^20^), bacteria translocation and proliferation can be exploited to deliver nanotherapeutics to hypoxic tumor tissue, poorly accessible to passively diffusing nanotherapeutics. Although it has been shown that chemotaxis^19^ and a combination of aerotaxis–magnetotaxis^20^ can be utilized to direct bacteria toward hypoxic tumor regions, the extent of penetration of chemotaxis‐defective *S*. Typhimurium VNP20009^30^ has not been explored before. Furthermore, metrics that facilitate quantitative assessment of spatial distribution of therapeutic agents in tumor tissue are lacking.

**Figure 2:**
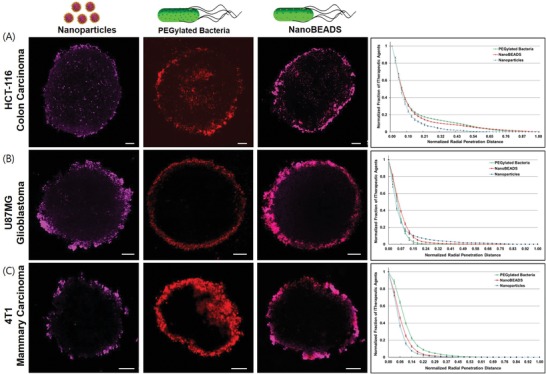
Intratumoral distribution of therapeutic agents. Confocal microscopy images and normalized radial distribution of the three therapeutic agents of PEGylated PLGA nanoparticles, PEGylated *S*. Typhimurium VNP20009, and PEGylated NanoBEADS in A) HCT‐116 human colon carcinoma, B) U87MG human glioblastoma, and C) 4T1 murine mammary carcinoma multicellular tumor spheroids. Radial locations were measured from the surface of the tumors. Bacteria are shown in red and nanoparticles are in purple. All scale bars are 100 µm.

We chose in vitro 3D multicellular tumor spheroids (MCTS) as the tumor model for the intratumoral transport assay, as they have been shown to effectively recapitulate key structural function and mass transport properties of avascular tumor tissue.^2^, ^31^, ^32^ Tumor spheroids were formed using HCT‐116 human colon carcinoma, U87MG human glioblastoma, or 4T1 murine mammary carcinoma cells. NanoBEADS, PEGylated bacteria, and PLGA nanoparticles were each incubated with the MCTS for the indicated duration (see the Experimental Section). PEGylated *S*. Typhimurium VNP20009 (hereafter referred to as PEGylated bacteria) and PEGylated PLGA nanoparticles (hereafter referred to as nanoparticles, see Experimental Section for preparation detail) serve as controls. The former is intended to elucidate the effect of nanoparticle load on bacteria intratumoral transport. The latter enables determination of the bacteria‐enhanced transport of nanoparticles. Upon completion of the experiments, tumors were washed, fixed, and sliced. Confocal microscopy images of tumor slices containing each of the three therapeutic agents were acquired and analyzed. **Figure** **2** shows the distinct differences in the dissemination patterns for different therapeutic agents and tumor types. Our confocal imaging results revealed that PLGA nanoparticles (visualized at 633 nm excitation wavelength with a 635–750 band‐pass filter) remain localized to the bacterial outer membrane (visualized at 543 nm excitation wavelength with a 553–624 band‐pass filter) during the intratumoral penetration of NanoBEADS (Figure S6, Supporting Information).

**Figure 3:**
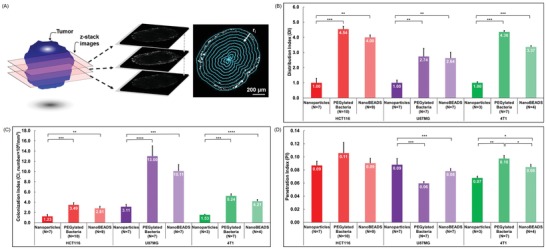
Quantitation of the intratumoral transport of therapeutic agents. A) To compute the intratumoral retention and transport metrics, confocal microscopy images of 40 µm thick tumor slices were segmented into 10 µm thick ring elements located at radial locations of *r_i_* from the surface of the tumor. B) Intratumoral distribution index, C) colonization index, and D) penetration index for PEGylated PLGA nanoparticles, PEGylated *S*. Typhimurium VNP20009, and PEGylated NanoBEADS through in vitro 3D tumor spheroid models of HCT116 (colon cancer), U87MG (brain cancer), and 4T1 (breast cancer). Each NanoBEADS agent carries an average of 22 nanoparticles, thus all NanoBEADS index values should be multiplied by 22 for comparing nanoparticle and NanoBEADS cases. Each data point represents the mean ± standard error and the number of independent replicates for each experiment is shown on the plot. *p value < 0.05; **p value < 0.01; ***p value < 0.001, ****p value < 0.0001.

We next wanted to develop metrics that describe both the quantity and spatial distribution of the three therapeutic agents within the tumor tissue and enable an objective comparison of their intratumoral transport performance. In the field of drug delivery, therapeutic distribution is described by parameters such as volume of distribution, plasma disappearance half‐life, and half‐life for clearance from the body.^33^ These parameters capture overall drug distribution and provide crucial information related to toxicity and effective administration schedule; however, they provide limited information about spatial distribution within tumors. For instance, a therapeutic agent might show an average higher concentration in tumor than in normal tissue (i.e., high volume of distribution), but if tissue penetration is poor, only cells in proximity of the blood vessels will be exposed to effective concentrations.^2^, ^34^ Thus, we developed three metrics to enable quantitative comparison of the therapeutic agent intratumoral transport. To compute each of these metrics, tumors were sliced into 40 µm thick sections, and each section was imaged using a laser scanning confocal microscope. Using a custom image processing routine (see the Supporting Information), each section was segmented into 10 µm thick ring elements located at radial locations of *r_i_* from the surface of the tumor (**Figure** **3**A). The first metric, penetration index (PI), provides a measure of the intratumoral penetration depth of the therapeutic agents toward the center of the tumor and is defined as

(1) $$\mathrm{PI}=\frac{\sum_{i=1}^{N}r_{i}\cdot n_{i}}{R_{\max}\cdot N_{\mathrm{MCTS}}}$$

where *r_i_* is the radial location of segment *i* measured from the tumor surface, *n_i_* is the number of the therapeutic agents within segment *i*, *R*_max_ is the maximum penetration distance (i.e., radius of the tumor spheroid), and *N*_MCTS_ is the total number of the therapeutic agents detected within the entire MCTS slice. The PI value ranges from 0 to 1, wherein a value of 1 indicates that all of the therapeutic agents travelled to the center of the tumor and 0 indicates that all of the therapeutic agents remained at the periphery of the tumor. The colonization index (CI) quantifies the number density of therapeutic agents within a given tumor and is defined as

(2) $$\mathrm{CI}\;=\;\frac{N_{\mathrm{MCTS}}}{V_{\mathrm{MCTS}}}$$

where *V*_MCTS_ is the entire MCTS slice. CI value signifies the tumor retention of the therapeutic agent of interest without consideration of its spatial distribution. Finally, the distribution index (DI) represents a composite normalized index of penetration and colonization, defined as

(3) $$\mathrm{DI}\;=\;\frac{\mathrm{PI}\;\times \;\mathrm{CI}}{{\left(\mathrm{PI}\;\times \;\mathrm{CI}\right)}_{\mathrm{NP}}}$$

where (PI × CI)_NP_ represents the intratumoral distribution of the PLGA nanoparticles. Thus, the DI provides a relative measure of intratumoral distribution efficacy compared with the passively diffusing nanoparticles. As shown in Figure 3B, the DI for NanoBEADS exceeds that of nanoparticles by 4.00‐fold, 2.64‐fold, and 3.37‐fold in HCT‐116 (colon cancer), U87MG (glioblastoma), and 4T1 (breast cancer) tumor spheroids, respectively. Given the colocalization of the nanoparticles and bacteria (Figure S6, Supporting Information), to minimize image processing errors (see Section II, Supporting Information), the DI of NanoBEADS was computed by measuring the fluorescence signal from the bacteria component of the system. Considering that an average of 22 nanoparticles are carried by each NanoBEADS agent, conjugation of nanoparticles with bacteria enhanced the transport efficacy of nanoparticles by ≈58–88 fold, compared to their passively diffusing counterparts. PEGylated bacteria had the highest DI in all tumors, although the differences between the DI values of PEGylated bacteria and NanoBEADS were not statistically significant. The 4.54‐fold and 4.36‐fold difference in the DI of PEGylated bacteria compared with nanoparticles in HCT‐116 and 4T1 tumor spheroids were significantly higher than the 2.74‐fold increase observed in U87MG tumor spheroids. Investigating the performance of therapeutic agent with respect to PI and CI separately further elucidates the different dissemination patterns due to dissimilar tumor properties (Figure 3C,D). In all three tumor types, the CI for PEGylated bacteria was the highest, closely followed by the CI for NanoBEADS. The CI values for nanoparticles were markedly lower across all the tumor types. The significantly higher CI for the bacteria and NanoBEADS is attributed to the higher initial entrapment and also proliferation within the tumor spheroid, which are the beneficial attributes of using bacteria as nanoparticle delivery vectors. The slightly lower CI of NanoBEADS could be due to their lower growth rate compared with the PEGylated bacteria. Contrary to the consistent CI trends across tumor types, the observed PI trends were tumor‐type dependent. For HCT‐116, there were no statistically significant differences in PI for PEGylated bacteria, NanoBEADS, and nanoparticles. For U87MG tumors, nanoparticles had the highest PI value, closely followed by NanoBEADS and PEGylated bacteria, with no statistically significant difference between the two. Finally, the PI trend in 4T1 tumors showed significant differences across the three therapeutic agents with PI values 42% and 14% higher for the PEGylated bacteria and the NanoBEADS, respectively, compared with the nanoparticles. These indexes corroborate the radial distribution profiles of the therapeutic agents within the tumors (Figure 2; Figure S7, Supporting Information). In HCT‐116 and 4T1 tumors, the distribution of nanoparticles within the tumor tissue drop precipitously with distance, whereas a more gradual decay is observed for PEGylated bacteria and NanoBEADS. By contrast, the radial distributions of the three therapeutic agent types seem to be comparable in the U87MG tumors, with nanoparticles performing slightly better than bacteria and NanoBEADS. Differences in the PI trends of the three tumor types suggest that the penetration differences might be due to dissimilar microenvironment properties (e.g., cell–cell adhesion strength or extracellular matrix density) between the tumors,^3^ as all three tumor types had similar cell densities (Figure S8, Supporting Information). To assess the differences in the extracellular environment of the tumor spheroids, the collagen content of the tumors was analyzed by immunofluorescence staining (Figure S9, Supporting Information). The HCT‐116 had the least amount of collagen (0.01%), followed by 4T1 (2.75%) and U87MG (13.24%). The markedly higher collagen content of the U87 tumors confirms our hypothesis that the denser microenvironment in this tumor type inhibits the intratumoral transport of the bacteria‐based therapeutic agents, whereas the smaller‐sized nanoparticles showed a slight advantage in penetrating the U87MG tumors. This assertion is consistent with prior reports in the literature showing that high collagen content inhibits diffusive transport of larger molecular weight therapeutics.^35^, ^36^ Altogether, the higher CI of bacteria‐based therapeutic agents dominates the microenvironment‐dependent PI trends, and overall more effective DI can always be achieved with bacteria irrespective of the tumor‐type. We have previously shown that *S*. Typhimurium VNP20009 is poorly motile and lacks chemotaxis.^30^ Thus, we show that bacteria enable significant transport enhancement through intercellular self‐replication and autonomous translocation, without the need for any externally applied driving force (e.g., magnetotaxis or chemotaxis). This assertion is consistent with findings in the literature on the mechanism of progression of bacteria in sub‐micrometer constrictions.^37^

### 2.5 Mechanism of the Penetration of Bacteria in 3D Tumor Spheroids

**Figure 4:**
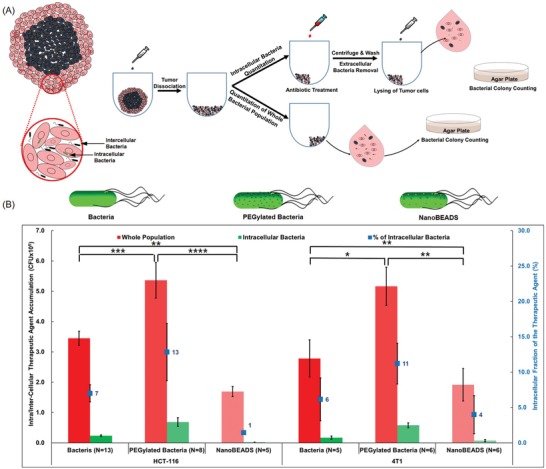
Mechanism of bacteria intratumoral transport. A) Experimental work flow for quantitating the whole (i.e., intracellular and intercellular) population and the intracellular subpopulation of bacteria and NanoBEADS. B) The whole population and the intracellular subpopulation of the unmodified bacteria, PEGylated bacteria, and NanoBEADS in 3D tumor spheroid models of HCT116 (colon cancer) and 4T1 (breast cancer). The percentage of intracellular population is shown on the second axis (in blue). Each data point represents the mean ± standard error and the number of independent replicates for each experiment is shown on the plot. *p value < 0.05; **p value < 0.01; ***p value < 0.001, ****p value < 0.0001.

Although various strains of Gram‐negative and Gram‐positive bacteria are being explored as direct oncolytic agents (for a complete review of bacteria‐based cancer therapy, refer to refs. 16, 17, 18), the mechanism of intratumoral translocation of bacteria remains poorly understood. The PI of PEGylated bacteria in the denser U87MG (brain cancer) tumor was ≈54% of the PI for PEGylated bacteria in HCT‐116 (colon cancer) and ≈60% of that in 4T1 (breast cancer). Given our observation of the dependency of bacteria intratumoral penetration on tumor‐type and the potential role of the tumor microenvironment, we hypothesized that the dominant mode of bacterial penetration is through intercellular translocation rather than intracellular invasion. To test this hypothesis, the number of therapeutic agents translocating intracellularly and intercellularly in the tumor spheroids were quantified using the gentamicin protection assay (**Figure** **4**A). As shown in Figure 4B, ≈13% and 11% of the PEGylated bacteria population and 1% and 4% of PEGylated NanoBEADS, in HCT116 and 4T1 tumors respectively, penetrated via intracellular translocation (data for U87MG not shown as we were not able to homogenously dissociate these tumors using enzymatic digestion). These results indicate that intratumoral penetration primarily occurs via intercellular translocation and supports the notion that collagen content and microenvironment structure differences contribute to the PI trends reported in Figure 3D. Furthermore, PEGylated bacteria showed higher overall colonization as well as an increase in the number of intracellular bacteria compared to the unmodified bacteria. This could be due to the fact that the PEG coating facilitated enhanced intratumoral penetration and intercellular translocation.^38^, ^39^ The higher concentration of PEGylated bacteria within the tumors, combined with proliferation during the incubation period, increased the number of therapeutic agents that were interacting with the cancer cells in the 3D space and provided a greater probability for cell invasion by PEGylated bacteria, compared to the unmodified bacteria. The larger size of the NanoBEADS agents combined with their slower growth rate led to a very low intracellular uptake of these agents. The intracellular bacteria penetration of PEGylated bacteria and NanoBEADS compared with the unmodified bacteria was further investigated in a 2D invasion assay.

### 2.6 Invasion of Therapeutic Agents in 2D Cell Culture

**Figure 5:**
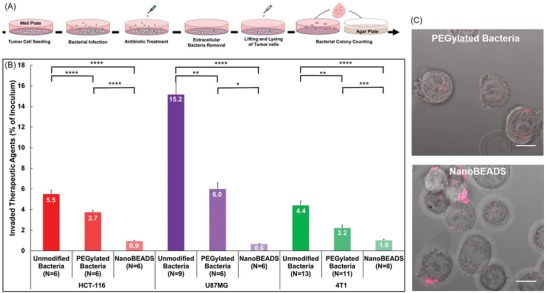
Effect of PEG coating and nanoparticle conjugation on bacterial invasion of cancer cells. A) Experimental design of the invasion assay. B) Percentage of each of the therapeutic agent inoculum invaded the HCT116 colon carcinoma, U87MG glioblastoma, and 4T1 mammary carcinoma cells. Each data point represents the mean ± standard error and the number of replicates for each experiment is shown on the plot. *p value < 0.05; **p value < 0.01; ***p value < 0.001, ****p value < 0.0001. C) Representative composite bright‐field and fluorescence microscopy images of invaded HCT‐116 cells. Scale bars are 10 µm.

To delineate the role of PEG coating and nanoparticle attachment on *S*. Typhimurium VNP20009 invasion of the cancer cells at the single cell scale, we carried out a series of 2D invasion assay experiments. Cellular uptake of unmodified *S*. Typhimurium VNP20009, PEGylated *S*. Typhimurium VNP20009, and NanoBEADS by HCT‐116 (colon cancer), U87MG (brain cancer), and 4T1 (breast cancer) cells was evaluated using the gentamicin protection assay at a multiplicity of infection (MOI) of 5. The results are shown in **Figure** **5**. Consistent with our 3D cellular uptake assay findings reported in Figure 4B, only a small fraction of the unmodified bacteria invaded the cancer cells. These results are also consistent with earlier reports of low levels of invasion of nonphagocytic epithelial cells by *S*. Typhimurium.^40^, ^41^ PEG coating and nanoparticle attachment both reduced bacterial invasion of the cancer cells. Compared with *S*. Typhimurium VNP20009, a smaller fraction of PEGylated bacteria and an even smaller fraction of the NanoBEADS agents were internalized. This trend was consistently observed across the cancer cell lines. Invasion of epithelial cells by *Salmonella enterica* serotype Typhimurium is mediated by the type III secretion system (T3SS) encoded by the *Salmonella* pathogenicity island‐1 (SPI1).^42^ T3SS‐independent invasion mechanisms that rely on outer membrane proteins, identified as *Salmonella* invasins, have recently been discovered,^43^, ^44^ although it is presently believed that that SP1 T3SS‐mediated process is the dominant mode of entry into nonphagocytic cells.^41^ The T3SS‐mediated invasion is described as a “trigger mechanism” wherein a molecular syringe directly delivers a cohort of virulence effector proteins into host cells which causes dramatic cytoskeletal rearrangement and membrane ruffle formation that results in bacterial internalization.^45^ By contrast, in outer membrane protein‐mediated invasion, invading bacteria utilize a “zipper” mechanism, or “receptor‐mediated entry,” wherein specific ligands (invasins) bind to host cell surface receptors with minimal cytoskeletal rearrangement.^46^ Considering these invasion mechanisms, we attribute the smaller fraction of the internalized PEGylated bacteria and NanoBEADS to the steric hindrance due to the PEG coating and nanoparticle attachment on the bacterial outer membrane. Increased hydrodynamic distance or spacing between the bacterial outer membrane and the tumor cell, due to the PEG chain length or particles attached on the bacteria, hinders engagement of the bacterial invasion machinery,^47^ and thus a lower number of therapeutic agents invade the cancer cells. Although on one hand, these bacterial modifications impede invasion of the tumor cells, on the other hand, they facilitate enhanced tumor uptake (Figure 4B). This phenomena, known as the PEG dilemma, is a well‐recognized challenge in drug delivery.^38^, ^39^ We expect that our bacteria‐based therapeutic agents can uniquely overcome this dilemma, as the PEG coating will facilitate enhanced initial uptake and intratumoral transport, but over time, bacterial proliferation leads to erosion of the PEG coating and enhanced cellular uptake (Figure 5B).

### 2.7 Intratumoral Distribution of the Therapeutic Agents In Vivo

**Figure 6:**
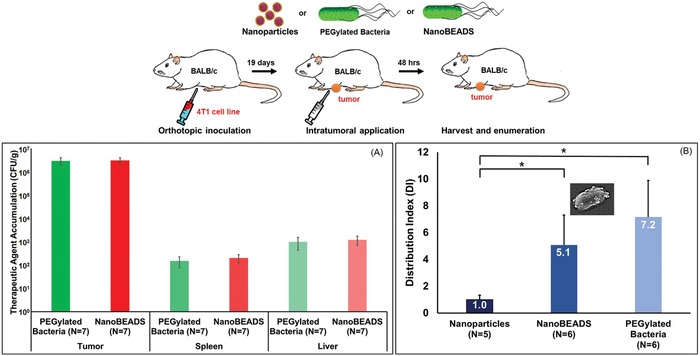
Biodistribution of the therapeutic agents. A) PEGylated bacteria and NanoBEADS concentration in 4T1 mammary tumors, spleens, and livers of BALB/c mice 48 h after intratumoral injection. B) Distribution index (DI) of the three therapeutic agents—PLGA nanoparticles, NanoBEADS, and PEGylated bacteria in 4T1 tumors. Each NanoBEADS agent carries an average of 22 nanoparticles, thus, it enhances the intratumoral transport of nanoparticles by up to ≈100‐fold. Each data point represents the mean ± standard error and the number of replicates for each experiment is shown on the plot. *p value < 0.05.

Effective tumor therapy requires delivery of therapeutics to distal malignant cells at lethal concentrations. Poor drug distribution necessitates multiple dosing and may contribute to the emergence of drug resistance.^10^ Thus, we investigated the spatial distribution of the three therapeutic agents, PLGA nanoparticles, PEGylated bacteria, and NanoBEADS, in an orthotopic model of breast cancer to evaluate whether the bacteria‐mediated enhanced intratumoral transport of nanoparticles that we observed in vitro (reported in Figure 3) is conserved in vivo. To this end, female BALB/c mice, bearing 4T1 mammary breast cancer tumors, were randomized into 4 groups of 7 mice. Two groups were intratumorally injected with 100 µL of PEGylated bacteria or NanoBEADS to a final concentration of 8.0 × 10^5^ CFU mL^−1^ in Dulbecco's phosphate buffered saline (DPBS). In the third group, PLGA nanoparticles with a final concentration of 1.8 × 10^7^ nanoparticles mL^−1^ in DPBS were used as the reference (or baseline), and the fourth group served as control. Nanoparticles were introduced at a higher concentration to match the number of nanoparticles that were introduced in the NanoBEADS case (≈22 nanoparticles per bacterium). After 48 h, tumors, livers, and spleens were harvested, and the extent of colonization of the bacteria‐based therapeutic agents was evaluated. Both therapeutic agents preferentially colonized the tumors, as compared with liver and spleen. We observed three orders of magnitude (three‐log) greater bacterial colonization in tumor compared to the liver and spleen (**Figure** **6**A). This is consistent with prior reports in the literature demonstrating ≈1000‐fold higher accumulation of *S*. Typhimurium in tumor sites compared to normal tissue.^11^, ^48^ There was no statistically significant difference between the number of PEGylated bacteria and NanoBEADS quantified in each tissue type (Figure 6A). The histopathological assessment of the liver and spleen revealed no significant differences between the three groups and the control group, suggesting that PEGylated bacteria and NanoBEADS do not cause any liver or spleen damage within the timeframe of our experiments (Figures S10 and S11, Supporting Information). We then quantified the intratumoral penetration and retention metrics, PI, CI, and DI, for each of the three therapeutic agents (Figure S12, Supporting Information). As expected, the convection‐enhanced i.t. administration led to similar PI values for all the three therapeutic agents (Figure S13A, Supporting Information). However, a combination of better retention and self‐replication led to an order of magnitude higher CI for bacteria and NanoBEADS (Figure S13B, Supporting Information), even though nanoparticles were introduced at 22‐fold higher doses. The DI value for NanoBEADS exceeds that of nanoparticles by 5.1‐fold, which clearly demonstrates retention and distribution enhancement through bacterial conjugation of nanotherapeutics (Figure 6B). Given that on average 22 nanoparticles are attached to each NanoBEADS agent, a remarkable nanoparticle distribution enhancement of up to ≈100 fold is achieved in this case. Our results indicate that despite the convection‐enhanced intratumoral delivery of nanoparticles, NanoBEADS continue to outperform nanoparticles in intratumoral distribution due to superior retention and their innate ability to translocate within the tumor as they self‐replicate.

## 3 Conclusion

Efficacious delivery of nanomedicine to all malignant cells in adequate concentrations is strongly dependent on the nanotherapeutics extravascular transport properties. This important challenge in cancer treatment has received little attention up to now (for a recent review of the topic, refer to ref. 4). Moreover, there has been a burgeoning interest in microbial‐mediated cancer therapy as an alternative approach for hard to treat cancers.^18^ Thus far, bacteria monotherapy has not been successful, and bacteria therapy protocols rely on adjuvant radiation or chemotherapy, which lead to systemic exposure and the associated side effects. The NanoBEADS platform provides a simple, versatile, and clinically applicable strategy to enhance the efficacy of bacteria while at the same time augmenting the specificity and transport of nanoparticles into sites poorly accessible from circulation without the need for any externally applied driving force (e.g., magnetic field) or genetic modification of bacteria. We showed that nanoscale drug delivery vectors can be stably bound to the bacteria without toxicity or statistically significant impediment in the bacteria intratumoral transport properties. Thus, NanoBEADS agents robustly surpass the convection/diffusion limits, primarily through intercellular self‐replication and translocation, and effectively carry nanoscale loads. The transport quantitation metrics (PI, CI, and DI) defined herein could serve as a framework for the quantitative evaluation of the spatial distribution of other therapeutics within tissue and an added measure for evaluating the efficacy of new therapeutic compounds for the treatment of solid tumors.

Our findings offer exciting new opportunities for enhanced targeted delivery and interstitial distribution of nanotherapeutics, which may enhance efficacy (by better distribution) and reduce systemic toxicity (by better targeting which reduces the required dosage) of existing nanomedicine and facilitate delivery of highly effective therapy compounds with severe dose‐limiting toxicity. The NanoBEADS platform also provides new pathways for engineering new cancer theranostic concepts constructed using other nanoscale loads such as imaging contrast vectors or biosensors. Furthermore, the tumor‐targeting bacteria can be engineered such that NanoBEADS agents operate as a network of biohybrid microrobotic agents^49^, ^50^, ^51^ with controlled interactions among themselves and with their immediate environment for programmed cargo delivery.

## 4 Experimental Section

*Bacteria Culture*: All experiments in this study were performed using the tumor targeting Salmonella enterica serovar Typhimurium strain VNP20009^25^ (ATCC 202165, American Type Culture Collection, Manassas, VA). Bacteria were transformed with a plasmid encoding a red fluorescent protein (RFP) for constitutive expression. The plasmid was constructed from BioBrick (iGEM Foundation, Cambridge, MA) parts. BBa_J04450, encoding mRFP1 expression, was assembled in the high copy number pSB1C3 vector conferring resistance to chloramphenicol. Lysogeny broth (LB; 1% w/v of tryptone, 1% w/v of NaCl, 0.5% w/v of yeast extract, supplemented with 35 µg mL^−1^ of chloramphenicol, pH 7.0) was inoculated with a single bacterial colony and shaken overnight at 37 °C and 100 rpm. Overnight bacteria culture was diluted 100‐fold in LB supplemented with chloramphenicol and shaken at 37 °C and 100 rpm until the optical density at 600 nm (OD_600_) reached 1.0. A 2 mL aliquot of the culture was then centrifuged at 1700 × g for 5 min at room temperature and suspended in 1 mL of freshly prepared motility buffer (MB; 6.4 × 10^−3^ m K_2_HPO_4_, 3.5 × 10^−3^ m KH_2_PO_4_, 0.1 × 10^−3^ m ethylenediaminetetraacetic acid (EDTA), 1 × 10^−6^ m L‐methionine, 10 × 10^−3^ m DL‐lactate, 2 × 10^−3^ m MgSO_4_, 2 × 10^−3^ m CaCl_2_, pH 7.0) and washed once more in MB prior to use in the biomanufacturing of the NanoBEADS.

*Nanoparticle Preparation*: The method used for nanoprecipitation of PLGA nanoparticles was modified from a method previously described by Niu et al.^24^ The biodegradable copolymer, PLGA (*M*_w_: 25 000 g mol^−1^, 50:50 lactic acid:glycolic acid, acid end‐capped, Akina Inc. PolySciTech, West Lafayette, IN) was used as received. PLGA was dissolved in dimethylformamide (DMF, spectrophotometric grade) at a concentration of 22.22 mg mL^−1^ and kept without any agitation for 30 min to wet the polymer and partly dissolve it before being sonicated (Branson 2510 Ultrasonic Cleaner, 100 W) for an additional 30 min at room temperature. Although minor heating occurred during sonication, the temperature was kept below 30 °C. During this time, Pluronic F127 (Sigma‐Aldrich, St. Louis, MO) was dissolved in deionized water at a concentration of 5 mg mL^−1^ by sonicating for 30 min first before being magnetically stirred at 600 rpm for an additional 30 min to reduce the bubbles that formed on the surface. Separately, the fluorophore 6,13‐bis(triisopropylsilylethynyl) pentacene (Sigma‐Aldrich, St. Louis, MO) was dissolved in tetrahydrofuran (THF, anhydrous and uninhibited, >99.9%, Sigma‐Aldrich, St. Louis, MO) at a concentration of 3.05 mg mL^−1^, by vortex mixing. The PLGA in DMF was then combined with the TIPS pentacene in THF at a volume ratio of 9:1 DMF:THF. This mixture had a final PLGA concentration of 20 mg mL^−1^ and a TIPS pentacene concentration of 0.305 mg mL^−1^ for a targeted TIPS pentacene loading of 1.5 wt% with respect to PLGA. To form the particles, 1 mL of the PLGA/TIPS pentacene solution was added drop‐wise (30 mL h^−1^) to the aqueous solution of Pluronic F127 using a 5 mL glass syringe equipped with a 21 gauge beveled needle and connected to a syringe pump (New Era Pump Systems, Farmingdale, NY) while magnetically stirred at 600 rpm. The combined solution was left stirring for 5 h, covered to minimize photobleaching of the fluorophore, before being centrifuged in 50 mL centrifuge tubes at 4 °C and 22789 × *g* for 30 min. The supernatant was decanted, and the pellet was then resuspended in 20 mL of DPBS by 30 min of sonication and then filtered through a 0.45 µm nitrocellulose membrane. The particles were stored in solution at room temperature and further processed within 4 days for the studies reported herein.

*Nanoparticle Surface Functionalization by EDAC Coupling of Streptavidin‐Cy3*: Streptavidin‐Cy3 (Sigma‐Aldrich, St. Louis, MO) was bound to the surface of the TIPS pentacene‐loaded PLGA nanoparticles using the PolyLink Protein Coupling Kit (Polysciences, Warrington, PA). First, streptavidin‐Cy3 was diluted from the 1 mg mL^−1^ stock solution to 100 µg mL^−1^ using the coupling buffer in the kit. The 1‐ethyl‐3‐(3‐dimethylaminopropyl) carbodiimide (EDAC) was dissolved separately at 200 mg mL^−1^ in the coupling buffer. The EDAC was freshly prepared for each experiment and was not stored in solution because EDAC is labile in aqueous solutions especially in slightly acidic conditions,^52^ as was the case for the coupling buffer (pH 5.2). Each batch of nanoparticles (≈4.5 × 10^9^ nanoparticles) required 4 mg of EDAC powder suspended in 20 µL of coupling buffer. A 10 µL aliquot of the diluted streptavidin‐Cy3 solution, 20 µL of the EDAC solution, and an additional 170 µL of coupling buffer were mixed to form 200 µL of coupling solution per batch of nanoparticles to be coated. A 175 µL aliquot of the stock nanoparticle suspension in DPBS was added to a 1.5 mL centrifuge tube and centrifuged down to a pellet at 16 060 × *g* for 10 min. The supernatant was aspirated, and 200 µL of the coupling solution was added. The pellet of nanoparticles was resuspended by gentle pipetting up and down followed by short vortex mixing. The suspension was then left to incubate for 3 h on a vortex mixer at 500 rpm. After incubation, the suspension was centrifuged again at 16 060 × *g* for 10 min, and the supernatant was aspirated. This time the particles were resuspended in 50 µL of MB for NanoBEADS formation. Separately, 175 µL of the PLGA nanoparticle suspension (≈4.5 × 10^9^ NPs) in DPBS was spun down to a pellet using a centrifuge (16 060 × g for 10 min) and was resuspended in 200 µL of coupling buffer with 5 µg mL^−1^ of streptavidin‐Cy3 and 20 mg mL^−1^ EDAC in order to conjugate streptavidin to the nanoparticle surface. The carboxylate groups on the surface of the PLGA nanoparticles bound to the amine groups of the streptavidin to form a covalent amide bond. After 3 h of incubation, the suspension was centrifuged at 16 060 × *g* for 10 min to remove unbound streptavidin. For the nanoparticle‐only experiments, this final pellet was resuspended in 100 µL of MB for the invasion assay, or in 100 µL of specific cell growth medium for the intratumoral transport assays. Furthermore, to avoid inadvertent interactions between the streptavidin binding sites present on the surface of nanoparticles and the cancer cells per tumors, the nanoparticles were incubated with mPEG‐biotin (*M*_w_: 5000 g mol^−1^, Laysan Bio, Arab, AL) at 0.8 µg mL^−1^ in culturing media for 30 min to quench all streptavidin binding sites. The selection of PEG molecular weight was informed by prior works on the effect of PEG chain length on nanoparticle penetration, which demonstrates that PEG 5 kDa improves penetration of nanoparticles in extracellular spaces^53^ and optimally reduces plasma protein adsorption.^54^

*Characterization of the PLGA Nanoparticles*: A Malvern Zetasizer Nano‐ZS was used to measure the hydrodynamic diameter and the zeta potential of the nanoparticles at 25 °C. DLS measurements of the size were conducted in DPBS diluted about 60‐fold. Immediately after fabrication and before surface functionalization, the intensity‐average hydrodynamic diameter of the particles was 120 ± 6 nm with the polydispersity index of 0.11, suggesting a narrow size distribution, as shown in Figure S1 (Supporting Information). The zeta potential of the particles was measured as a function of the incubation time with streptavidin‐Cy3 and EDAC. The liquid phase conductivity at which the zeta potentials were measured for this incubation study was ≈0.5 mS cm^−1^. Immediately after the nanoparticle fabrication, the zeta potential was −26 ± 5 mV. After incubation with streptavidin‐Cy3 for 3 h, the zeta potential rose to +31 ± 6 mV. The TIPS pentacene loading in the PLGA nanoparticles was measured using UV–Vis spectroscopy (Thermo Scientific, Evolution 300). Freeze‐dried particles were dissolved in mixtures of THF:DMF of 1:9 v/v at a known concentration to release the TIPS pentacene into solution. The sample absorbance was compared to a calibration curve of TIPS pentacene at *l* = 641 nm in the same solvent system to back‐calculate the TIPS pentacene concentration in solution and, hence, the loading. The final measured loading of TIPS pentacene in the PLGA nanoparticles was 0.9 wt% with respect to the mass of PLGA. This corresponds to an encapsulation efficiency of ≈60%.

*NanoBEADS Biomanufacturing and Characterization: S*. Typhimurium VNP20009 bacteria were suspended in 1 mL of MB to a final concentration of 4.5 × 10^8^ CFU mL^−1^ and were incubated with 10 µg mL^−1^ of rabbit polyclonal anti‐*S*. Typhimurium antibody conjugated with biotin (Thermo Scientific, Waltham, MA, USA) for 60 min at 500 rpm on a vortex mixer. Free antibody was removed by centrifugation at 1700 × g for 5 min. The bacterial suspension was concentrated in 0.5 mL of MB at 9.0 × 10^8^ CFU mL^−1^. 50 µL of streptavidin‐Cy3 coated PLGA nanoparticles (≈4.5 × 10^9^ NPs) in MB, prepared as described earlier, was combined with 50 µL of biotinylated antibody‐coated bacteria. The NanoBEADS agents were assembled through streptavidin–biotin binding via vortex mixing of the streptavidin‐coated PLGA particles and biotinylated antibody‐coated bacteria for 30 min. The NanoBEADS suspension was centrifuged through a 0.8 µm size centrifugal filter (Sartorius Vivaclear, Elk Grove, IL) at 1700 × g for 1 min to selectively remove unbound nanoparticles. The NanoBEADS on the filter membrane were collected by resuspending in the appropriate medium for each experiment. In order to avoid inadvertent interaction between cancer cells/tumors and unoccupied streptavidin binding sites present on the surface of nanoparticles, NanoBEADS were incubated with mPEG‐biotin (*M*_w_: 5000 g mol^−1^, Laysan Bio, Arab, AL) at 0.8 µg mL^−1^ in culturing media for 30 min to quench unbound streptavidin binding sites. In order to provide a fair comparison of bacteria to the NanoBEADS, PEGylated bacteria were prepared by coating the bacteria with biotinylated antibody at 10 µg mL^−1^ for 1 h, followed by streptavidin–cy3 conjugation at 5 µg mL^−1^ for 30 min, and biotinylated‐PEG coating at 0.8 µg mL^−1^ for 30 min. The number of PLGA nanoparticles carried by each NanoBEADS agent was determined using a field emission scanning electron microscope (FE‐SEM). To acquire the SEM images, the NanoBEADS were fixed overnight in a solution of 2.5% (v/v) glutaraldehyde in DPBS at 4 °C. Several 2 µL droplets of the fixed suspension were then transferred onto a glass slide and incubated for 10 min. The slide was then rinsed with deionized water and dried overnight. The samples were sputter coated with gold/palladium prior to imaging. High‐resolution SEM images were obtained using a Leo Zeiss FE‐SEM at a beam voltage of 5 kV, and a working distance of <8.2 mm. A minimum of 80 NanoBEADS agents were evaluated in each case to determine the number of PLGA nanoparticles attached to the bacterial outer membrane.

*Mammalian Cell Culture*: Three cell lines were used for the experiments—Human colon cancer (HCT‐116, ATCC CCL‐247), human brain cancer (U87MG, ATCC HTB‐14), and murine mammary carcinoma (4T1, ATCC CRL‐2539). The culturing medium requirements for all cell lines are shown in Table S1 (Supporting Information). For all cell lines, cells were seeded in a T‐25 flask with the corresponding culturing medium and incubated at 37 °C with 5% CO_2_. When the culture became ≈80% confluent, cells were lifted with 1 mL of 0.25% Trypsin‐EDTA solution (ATCC, Manassas, USA) and the cell density was estimated using a hemocytometer. The required number of the cells was transferred for each experiment as specified below.

*Tumor Spheroid Formation*: A previously established method of 3D multicellular tumor spheroid formation in ultralow adhesion well plates was adopted.^55^ Briefly, 15 000 cancer cells suspended in 200 µL of culture medium were seeded in each well of a 96 well ultralow attachment plate (Corning Inc., Corning, NY). The well plates were centrifuged at 1000 × g for 10 min and incubated at 37 °C and 5% CO_2_ until tumor spheroids grew to ≈1 mm in diameter in 5–7 days. Culture medium was changed every two days during the tumor growth period.

*Intratumoral Penetration of Nanoparticles, Bacteria, and NanoBEADS through In Vitro Tumor Spheroids*: To quantify the intratumoral penetration of each agent, multicellular tumor spheroids were separately infected with PLGA nanoparticles, PEGylated bacteria, or NanoBEADS. The number of therapeutic agents was maintained constant for all experiments as 1.8 × 10^10^ for nanoparticles, 1.8 × 10^8^ colony forming units (CFU) for bacteria, and 1.8 × 10^8^ CFU for NanoBEADS suspended in 100 µL of the corresponding cell culture medium. These concentrations were selected such that comparable numbers of PLGA nanoparticles were introduced in all cases. The tumor spheroids with therapeutic agents were incubated for 12 h at 37 °C with 5% CO_2_ on a vortex mixer operated at 500 rpm to prevent sedimentation of the therapeutic agents. Once the incubation period was completed, the tumors were rinsed with DPBS three times and prepared for imaging.

*Quantification of Bacterial Intra/Intercellular Penetration through the In Vitro Tumor Spheroids*: In order to elucidate the dominant route of tumor penetration, the number of therapeutic agents translocated intracellularly and intercellularly within the tumor spheroids was quantified. Tumor spheroids (≈1 mm diameter) were infected with 1.8 × 10^8^ CFU of bacteria, and an equivalent number of NanoBEADS agents suspended in 100 µL of the corresponding cell culture medium and incubated at 37 °C with 5% CO_2_ on a vortex mixer set to 500 rpm for 12 h. Subsequently, the tumors were rinsed with DPBS at least three times to wash away the bacteria loosely associated on the periphery of the tumor spheroid. Tumor spheroids were dissociated by incubation in 1 × Accumax (Innovative Cell Technologies, Inc., San Diego, CA) for 30 min. The resulting single‐cell suspensions were divided into halves for quantifying either the total number of bacteria or the number of intracellular bacteria. For intracellular bacteria quantitation, the dissociated cells were incubated with 50 µg mL^−1^ gentamicin sulfate for 1 h to kill the extracellular bacteria. The suspension was centrifuged at 900 × g for 2 min, and the supernatant was discarded. The pellet was suspended in 1% Triton X‐100 for 10 min to lyse the cell. The final suspension was sonicated (Branson 1510 Ultrasonic Cleaner, 100 W) for 15 s twice and plated on 1.5% LB agar plate. The total number of bacteria (intracellular and intercellular) was quantified using a similar procedure except that the antibiotic treatment step was eliminated. The number of intercellular bacteria was determined by subtracting the number of intracellular bacteria from the total number of bacteria.

*2D Invasion Assay of Bacteria and NanoBEADS*: Invasion assays were carried out using HCT116, U87MG, and 4T1 cells. Cells were seeded at 6 × 10^4^ cells per well in issue culture‐treated 12‐well plates and incubated at 37 °C with 5% CO_2_ overnight. The cells were then infected with the therapeutic agents (unmodified bacteria, PEGylated bacteria, or PEGylated NanoBEADS) at 3.0 × 10^5^ CFU per well, to achieve a MOI of 5:1. The therapeutic agents were incubated with the cells for 45 min at 37 °C with 5% CO_2_ to allow invasion. Afterward, the suspension was replaced with fresh cell culture medium with 50 µg mL^−1^ gentamicin sulfate and incubated for 1 h to kill extracellular bacteria, whereas intracellular bacteria were protected by the cell membrane. After a careful wash with DPBS, cells were treated with 0.25% trypsin‐EDTA (ATCC, Manassas, VA) for 10 min followed by 1% Triton X‐100 (Fisher Scientific, Pittsburgh, PA) for 10 min to detach and lyse the cells, respectively. Subsequently, the suspension was sonicated for 30 s to break up the clumps of bacteria, and the bacteria suspension was diluted to be plated for colony counting and determination of the number of intracellular bacteria.

*Image Acquisition and Processing*: The tumor spheroids were fixed in 4% paraformaldehyde in DPBS for 12 h at 4 °C and gently rinsed DPBS. The spheroids were transferred into cryomolds filled with a 1:1 (v/v) mixture of optimal cutting temperature (OCT) compounds and 60% w/v sucrose in deionized water. The tumor spheroids deposited in cryomold were stored at −20 °C for at least 30 min before cryosectioning. Using a cryotome, 40 µm thick slices were sectioned and transferred on the poly‐l‐lysine coated glass slide. A 10 µL droplet of deionized water was added onto each tumor slices, and a coverslip was placed on top. A Zeiss LSM 880 confocal laser scanning microscope was used to acquire images of the tissue slices through an LD C‐Apochromat 63 × 1.15 water‐immersion objective lens. TIPS pentacene‐loaded PLGA nanoparticles were visualized with a He/Ne laser at 633 nm excitation wavelength with a 635–750 nm band‐pass emission filter. The RFP‐expressing VNP20009 were visualized with a He/Ne laser at 543 nm excitation wavelength with a 553–624 band‐pass emission filter. A series of images at different depths (z‐stack) within a tumor slice were acquired using the Zeiss Zen 2 (black edition) software. Intratumoral penetration performance was quantified using a custom image processing algorithm, developed in MATLAB (see the Supporting Information for detail).

*Collagen Content Analysis*: Multicellular tumor spheroids were harvested, washed twice with DPBS, snap frozen in 1:1 (v/v) mixture of OCT compounds and 60% w/v sucrose, and stored at −80 °C for at least 30 min before cryosectioning. Cryosections of 10 µm thickness were transferred on poly‐l‐lysine coated glass slides, fixed in 4% paraformaldehyde in DPBS (pH 7.4) for 10 min at room temperature, gently rinsed with 2 mL of ice‐cold DPBS three times, and incubated with 5% normal goat serum for 30 min to block unspecific binding of the antibodies. Collagen staining was carried out by an overnight incubation with 20 µg mL^−1^ of rabbit polyclonal anti‐Collagen type I antibody (Abcam, Cambridge MA), followed by three washes with DPBS to remove unbound antibody and a subsequent 1 h incubation with 20 µg mL^−1^ of goat anti‐rabbit IgG H&L (Alexa Fluor 488) secondary antibody. After three washes with DPBS, nuclei were counterstained with NucBlue (Thermo Fisher Scientific, Waltham, MA) for 15 min. A droplet of mounting medium (Thermo Fisher Scientific, Waltham, MA) was added onto each tumor slices, and a coverslip was placed on top. A Zeiss LSM 880 confocal laser scanning microscope was used to acquire images of the tissue slices through an LD C‐Apochromat 40 × 1.2 water‐immersion objective lens. Image were imported into ImageJ, converted into binary images, and analyzed to determine the collagen content as the areal fraction of Alexa Fluor 488 staining.

*Syngeneic Tumor Model Experiment*: The Institutional Animal Care and Use Committee approved all experiments and the National Institute of Health guidelines for care and use of laboratory animals were strictly observed. Female 8–10 week‐old BALB/c mice were purchased from The Jackson Laboratories (Bar Harbor, ME) and were acclimated in cages for a week prior to tumor cell injection. 4T1 murine mammary carcinoma cells were cultured in Roswell Park Memorial Institute (RPMI)‐1640 medium supplemented with 10% fetal bovine serum, 100 units mL^−1^ penicillin, and 100 µg mL^−1^ streptomycin at 37 °C with 5% CO_2_. A 1.2 × 10^6^ aliquot of cells suspended in 100 µL DPBS was subcutaneously injected into the mammary fat pad of anesthetized mice. Weights and tumor size were documented twice per week, where tumor size was estimated using two perpendicular diameter measurements. The mice were euthanized if any of the following conditions were observed: 1) weight loss was more than 10% of the initial body weight, 2) the tumor grew larger than 1.4 cm diameter, or 3) clearly clinically moribund.

*Biodistribution and Histopathology*: PEGylated bacteria and NanoBEADS were prepared to a final concentration of 8.0 × 10^5^ CFU mL^−1^ in DPBS, whereas nanoparticles were prepared with a final concentration of 1.8 × 10^7^ nanoparticles mL^−1^ in DPBS. These concentrations were selected such that comparable numbers of PLGA nanoparticles were injected in all cases. Nineteen days after the tumor cell injection, the tumor‐bearing mice were randomized into 4 groups of 7 mice. 100 µL aliquots of the therapeutic agents in DPBS were administered through direct intratumoral (i.t.) injection. The same volume of DPBS was injected into the control group. Mice were euthanized 48 h postinjection. Tumor, liver, and spleen tissues were harvested and split into two equally sized pieces. One piece was used for confocal microscopy imaging and histopathology analysis. The PI, CI, and DI of each therapeutic agent category were computed from the confocal microscopy images where the point of injection is considered as the radial coordinate origin in PI calculations. The other halves of the harvested tumor, liver, and spleen tissue were weighed and homogenized in a known volume of DPBS with a disposable pestle system and diluted in DPBS prior to plating on 1.5% LB agar plates for bacterial colony counting. After 24–48 h incubation at 37 °C, the number of CFU were counted to determine the bacteria or NanoBEADS concentration (CFU g^−1^). Tissue samples for histopathology evaluation were fixed in 10% buffered formalin, paraffin embedded, sectioned at 5 µm, and stained with hematoxylin and eosin. Livers were graded by a board‐certified veterinary pathologist (S.L.C.) on a scale from 0–4 on the percentage of the sections affected by necrosis, extramedullary hematopoiesis, and inflammation. The individual scores were then summed to create a composite score.

*Statistical Analysis*: All the results shown are from at least three independent experiments. Data are expressed as means ± S.E. All statistical analyses were performed with OriginPro (OriginLab, Northampton, MA). A two‐sample *t*‐test was performed for pairwise comparisons. Data with more than two categories were analyzed using the one‐way analysis of variance method followed by Fisher's least significant difference test. *p* values less than 0.05 were considered significant.

## Conflict of Interest

The authors declare no conflict of interest.

## Supporting information

Supplementary
