# Supplementary material for: Nanoscale Bacteria‐Enabled Autonomous Drug Delivery System (NanoBEADS) Enhances Intratumoral Transport of Nanomedicine
Source: Adv Sci (Weinh). 2018 Dec 5;6(3):1801309. doi: 10.1002/advs.201801309 (PMC6364498; doi:10.1002/advs.201801309)
Supplement: Supplementary file 1 — Supplementary [file ADVS-6-1801309-s001.pdf]

Copyright WILEY-VCH Verlag GmbH & Co. KGaA, 69469 Weinheim, Germany, 2018.

ADVANCED SCIENCE
Open Access

Supporting Information

for *Adv. Sci.,* DOI: 10.1002/advs.201801309

Nanoscale Bacteria-Enabled Autonomous Drug Delivery System (NanoBEADS) Enhances Intratumoral Transport of Nanomedicine

*SeungBeum Suh, Ami Jo, Mahama A. Traore, Ying Zhan, Sheryl L. Coutermarsh-Ott, Veronica M. Ringel-Scaia, Irving C. Allen, Richey M. Davis, and Bahareh Behkam**

Supporting Information

Nanoscale Bacteria-Enabled Autonomous Drug Delivery System (NanoBEADS) Enhances Intratumoral Transport of Nanomedicine

*SeungBeum Suh, Ami Jo, Mahama A. Traore, Ying Zhan, Sheryl L. Coutermarsh-Ott, Veronica M. Ringel-Scaia, Irving C. Allen, Richey M. Davis, and Bahareh Behkam**

Dr. S. Suh, Dr. M. A. Traore, Y. Zhan, Prof. B. Behkam
Department of Mechanical Engineering
Virginia Tech
Blacksburg, VA 24061, USA
Email: behkam@vt.edu

Dr. A. Jo, Prof. R. M. Davis
Department of Chemical Engineering
Macromolecules Innovation Institute
Virginia Tech
Blacksburg, VA 24061, USA

Prof. S. L. Coutermarsh-Ott, V. M. Ringel-Scaia, Prof. I. C. Allen,
Department of Biomedical Sciences and Pathobiology
Virginia Tech
Blacksburg, VA 24061, USA

Prof. B. Behkam
Macromolecules Innovation Institute
School of Biomedical Engineering & Sciences
Virginia Tech
Blacksburg, VA 24061, USA

Keywords
bio-hybrid systems, tumor-targeting bacteria, *Salmonella enterica* serovar Typhimurium, bacteria-based therapies, intratumoral penetration, extravascular transport

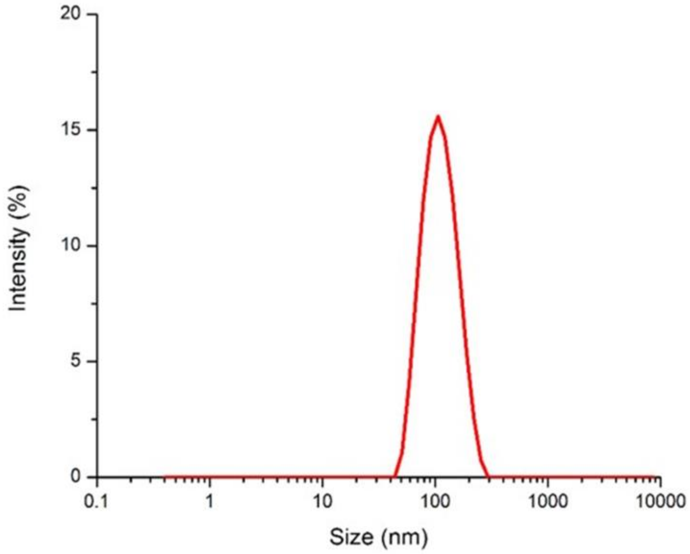

Figure S1. Dynamic Light Scattering Analysis. Representative intensity-size distribution plot of TIPS pentacene-loaded PLGA nanoparticles in PBS at a concentration of ~0.02 mg mL^{-1}. The polydispersity index (PDI)=0.11 suggests a narrow size distribution.

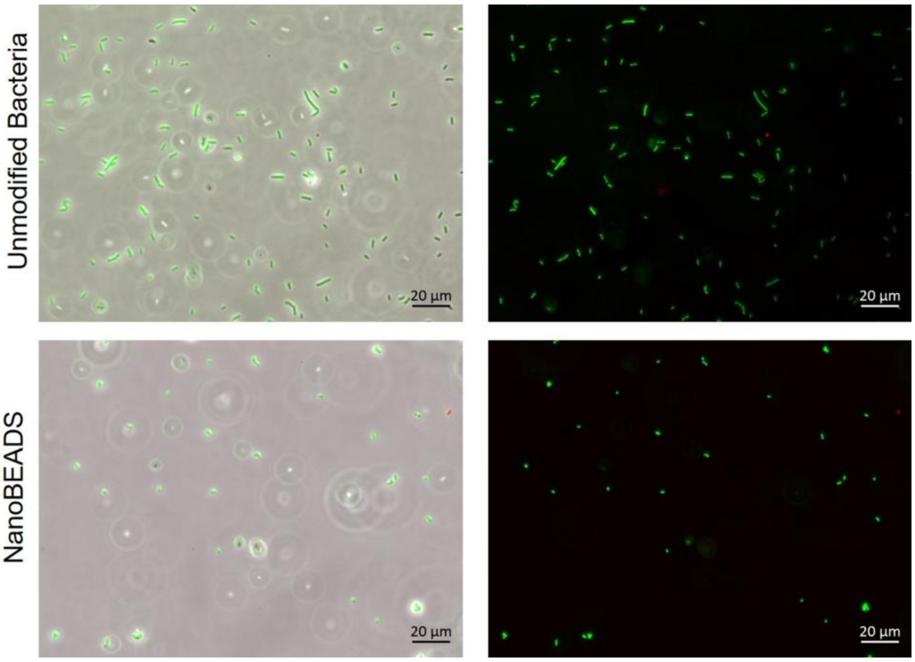

Figure S2. NanoBEADS Viability. Composite bright-field and fluorescence microscopy images of unmodified bacteria and NanoBEADS (left column). The fluorescence microscopy images (right column) show live bacteria (green) and dead bacteria (red). Conjugation of nanoparticles (22±14) to bacteria through biotin-streptavidin affinity bonds does not affect bacteria viability. All scale bars are 20 µm.

I. Supplementary Figures and Tables

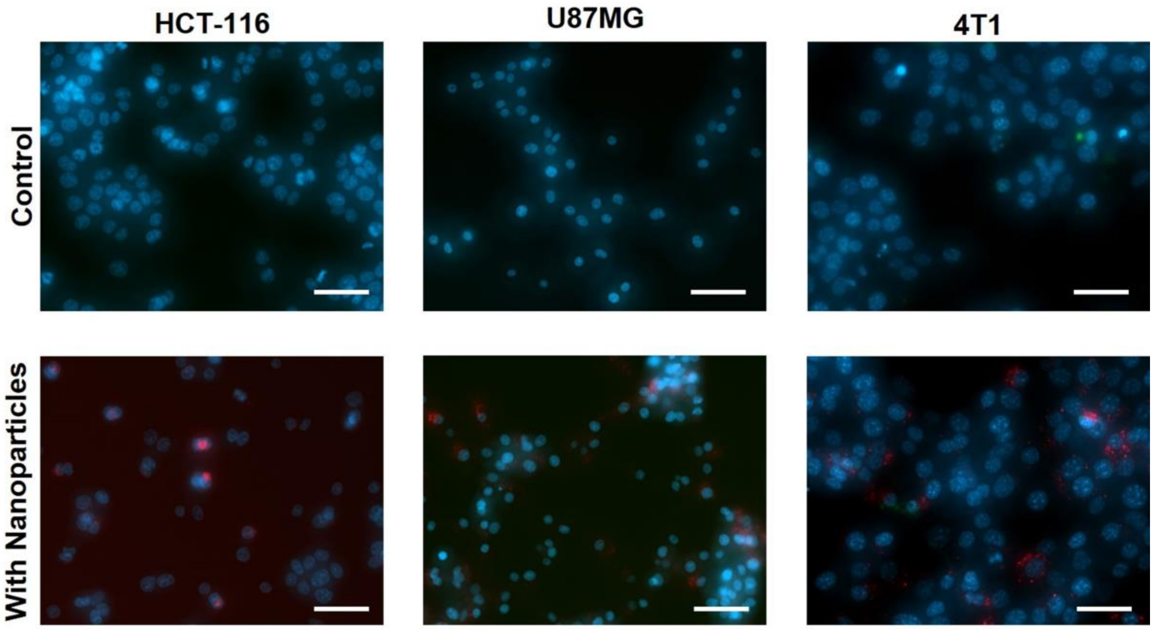

Figure S3. Cancer Cell Viability in Presence of TIPS-pentacene-loaded PLGA Nanoparticles. Composite fluorescence microscopy images show live cancer cells (blue) and dead cancer cells (green) in absence (control) and presence of 6×10^{8} mL^{-1} TIPS-pentacene-loaded PLGA Nanoparticles (red) after 12 hours of incubation. No dead cells were observed, suggesting that the nanoparticles do not affect cancer cell viability. All scale bars are 50 µm.

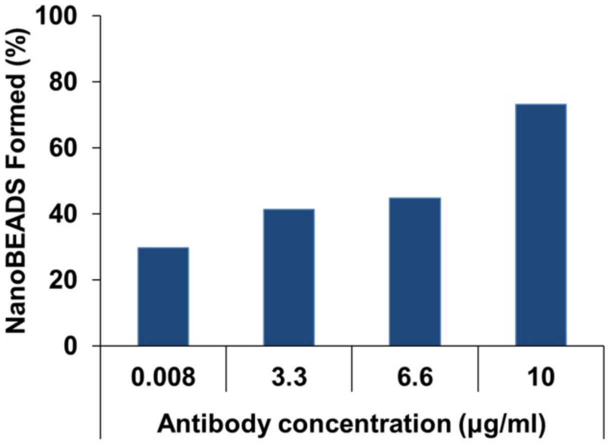

Figure S4. Effect of Antibody Concentration on NanoBEADS Formation. At nanoparticle to bacteria assembly ratio of 50:1, the fraction of bacteria with at least one nanoparticle attached steadily rose with increasing concentration of antibody till the maximum concentration of 10 µg mL^{-1}.

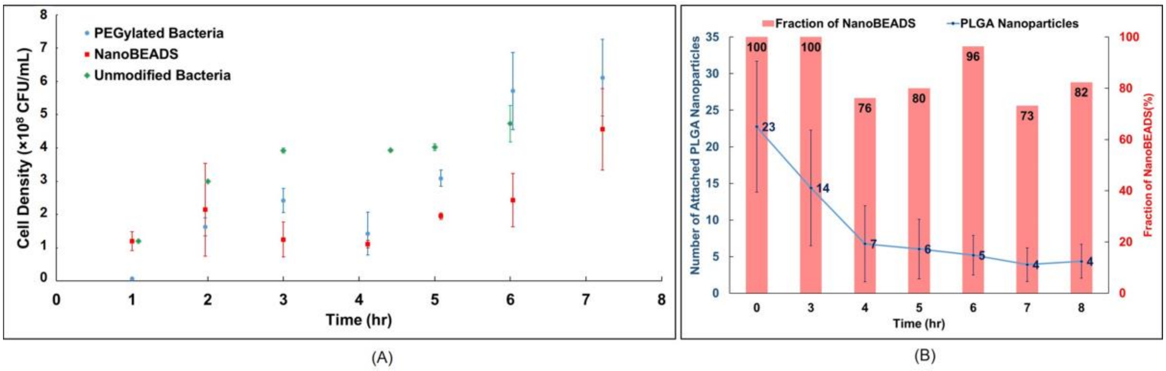

Figure S5. NanoBEADS Growth. (A) Effect of surface coating on bacteria growth. Bacteria growth rate decreases with addition of PEG coating or PLGA nanoparticles to the bacteria outer membrane. The doubling time of unmodified bacteria, PEGylated bacteria, and NanoBEADS in McCoy's 5A under ideal microbiological culturing conditions were measured to be 43, 58, and 121 minutes, respectively**. (B) Effect of NanoBEADS growth on attached PLGA nanoparticles.** As NanoBEADS agent undergo binary fission, the stably attached PLGA nanoparticles are divided amongst the daughter cells.

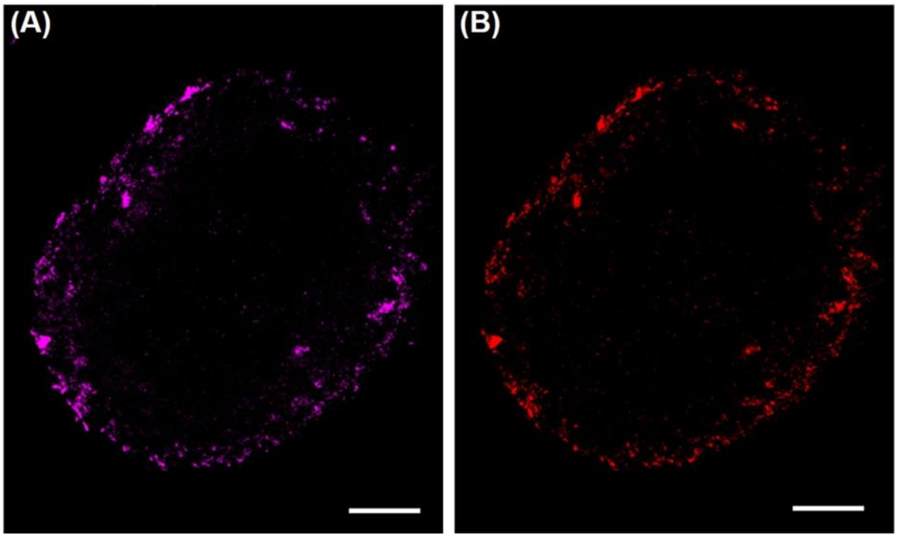

Figure S6. Co-localization of Bacteria and Nanoparticles in Tumors. Confocal microscopy images of NanoBEADS agents in HCT-116 tumors after 12 hours show that **(A)** PLGA nanoparticles (visualized at 633 nm excitation wavelength with a 635-750 band-pass filter) remain localized to **(B)** the bacteria outer membrane (visualized at 543 nm excitation wavelength with a 553-624 band-pass filter) during the intratumoral penetration of NanoBEADS. All scale bars are 100 µm.

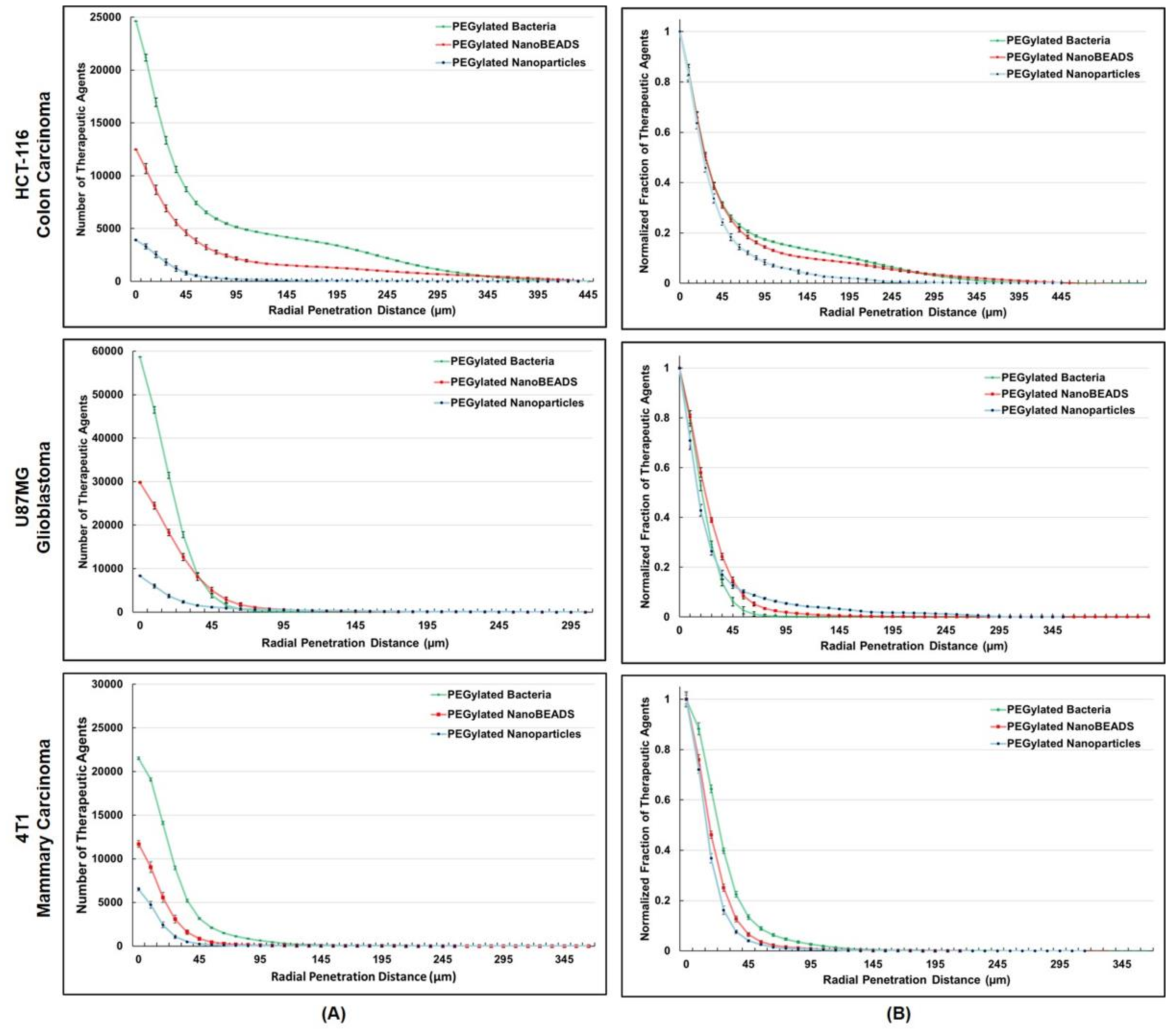

Figure S7. Radial distribution of Therapeutic Agents in Multicellular Tumor Spheroids. Absolute **(A)** and normalized **(B)** therapeutic agent numbers vs. radial location, measured from the surface of the tumors. For HCT-116 tumor spheroids, N=10 for PEGylated bacteria, N=9 for NanoBEADS, and N=7 for nanoparticles. For U87MG tumor spheroids, N=7 for PEGylated bacteria, N=7 for NanoBEADS, and N=7 for nanoparticles. For 4T1 tumor spheroids, N=7 for PEGylated bacteria, N=4 for NanoBEADS, and N=3 for nanoparticles.

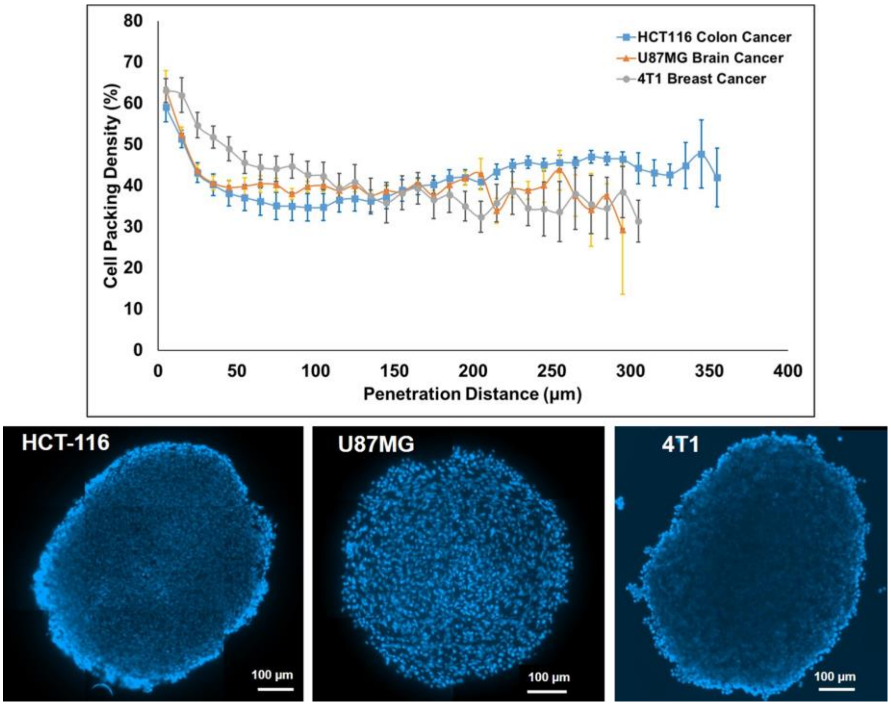

Figure S8. Cell Density in Tumor Spheroids. Confocal microscopy images of NucBlue stained HCT-116 human colon carcinoma, U87MG human glioblastoma, or 4T1 murine mammary carcinoma tumor slices were analyzed to determine the cell packing density in each tumor type. No significant difference in the cell packing density was observed.

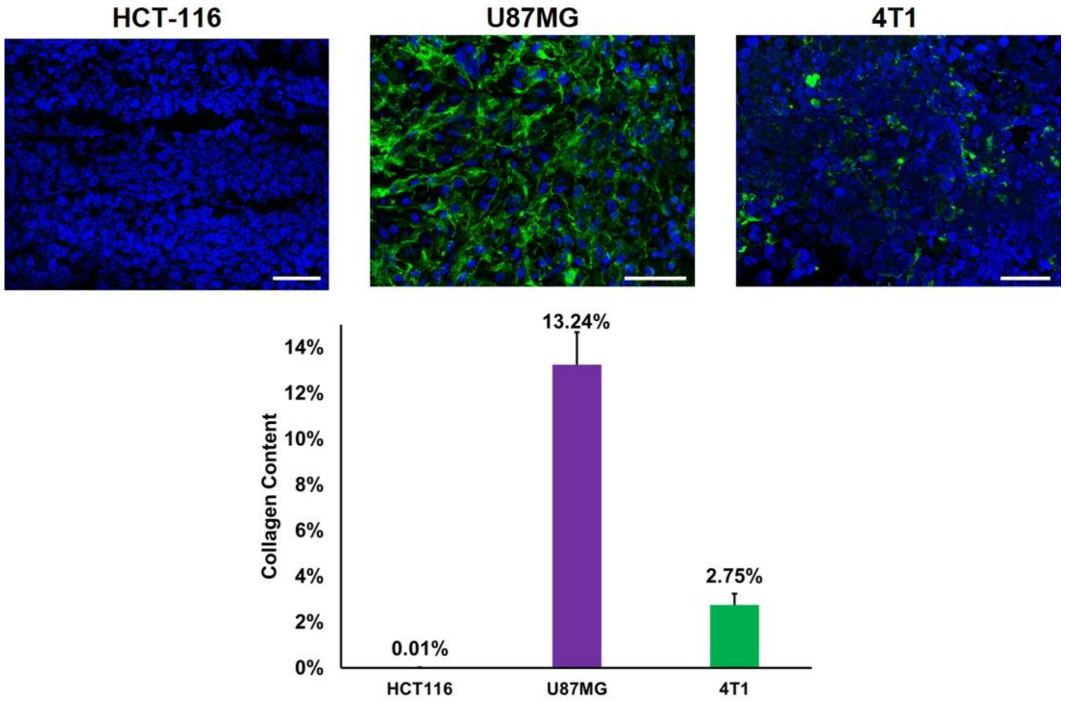

Figure S9. Collagen Content in Tumor Spheroids. Representative confocal microscopy images and quantification of collagen type I immunofluorescence staining in HCT-116 human colon carcinoma, U87MG human glioblastoma, or 4T1 murine mammary carcinoma tumor slices. Green, collagen I staining; blue, DAPI. All scale bars are 50 µm.

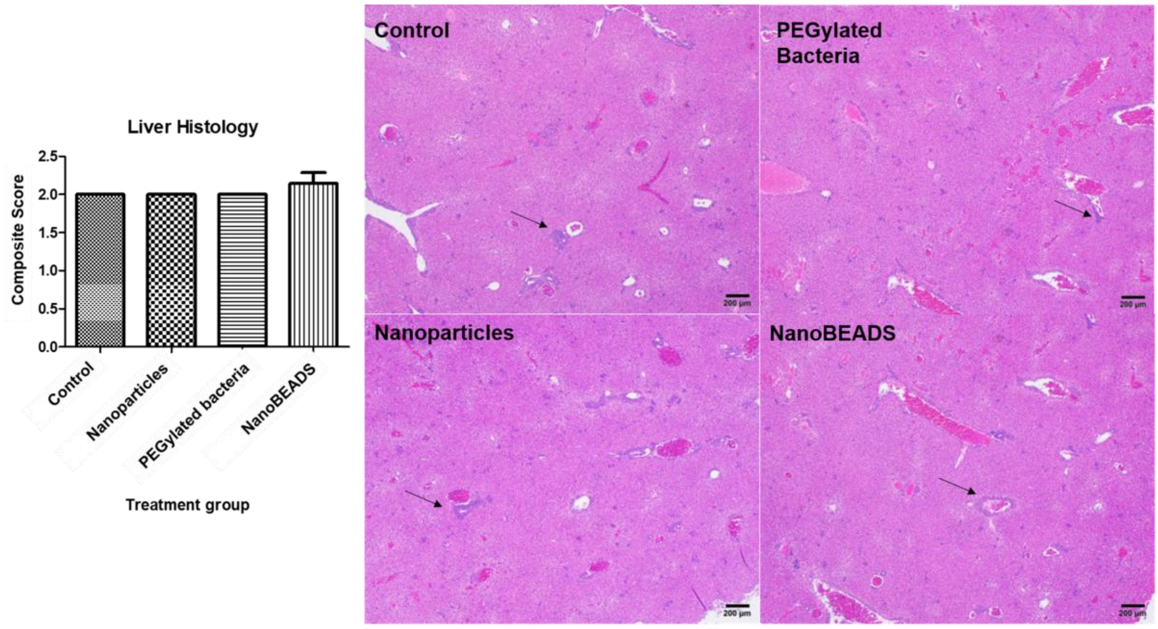

Figure S10. Histological Assessment of the Liver Tissue. Liver tissue samples (N=7 for each group) were graded on a scale from 0-4 on the percentage of the sections affected by necrosis, extramedullary hematopoiesis (EMH), and inflammation. The individual scores were then summed to create a composite score. There were no significant differences between the groups with regards to liver scores. Areas of hypercellularity (noted by arrows) are inflammation and/or EMH. All scale bars are 200 µm.

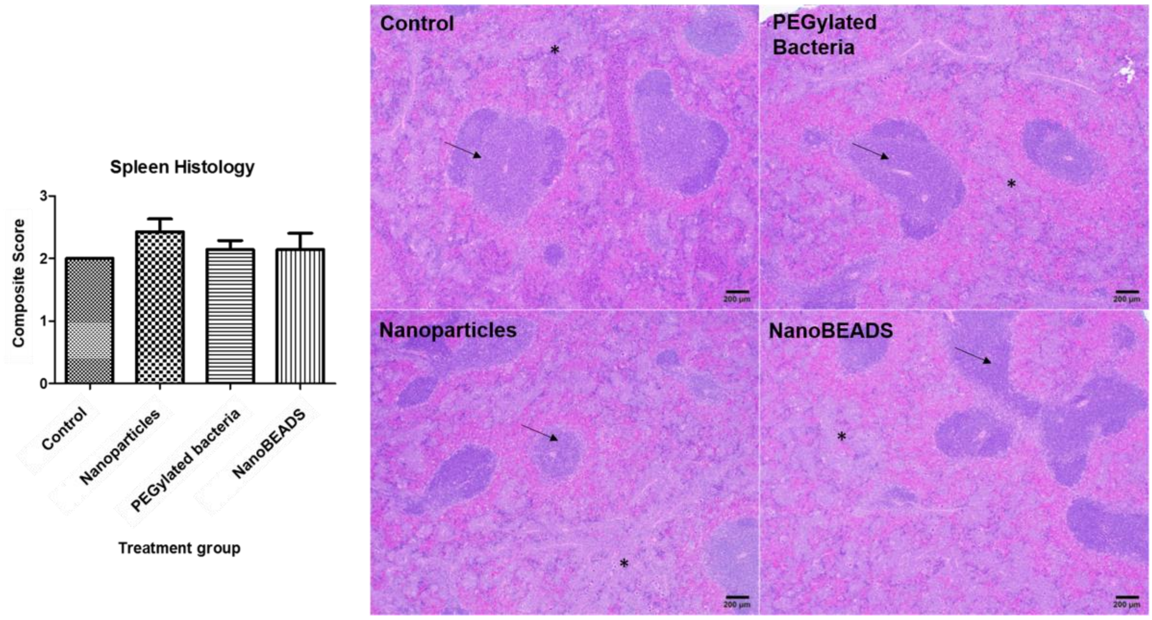

Figure S11. Histological Assessment of the Spleen Tissue. Spleen tissue samples (N=7 for each group) were graded on a scale from 0-4 on the percentage of the sections affected by necrosis, EMH, and inflammation. The individual scores were then summed to create a composite score. There were no significant differences between the groups with regards to spleen scores. The arrows are pointing to normal lymphoid tissue in the spleen and the asterisks are pointing to the pale EMH in the adjacent red pulp. All

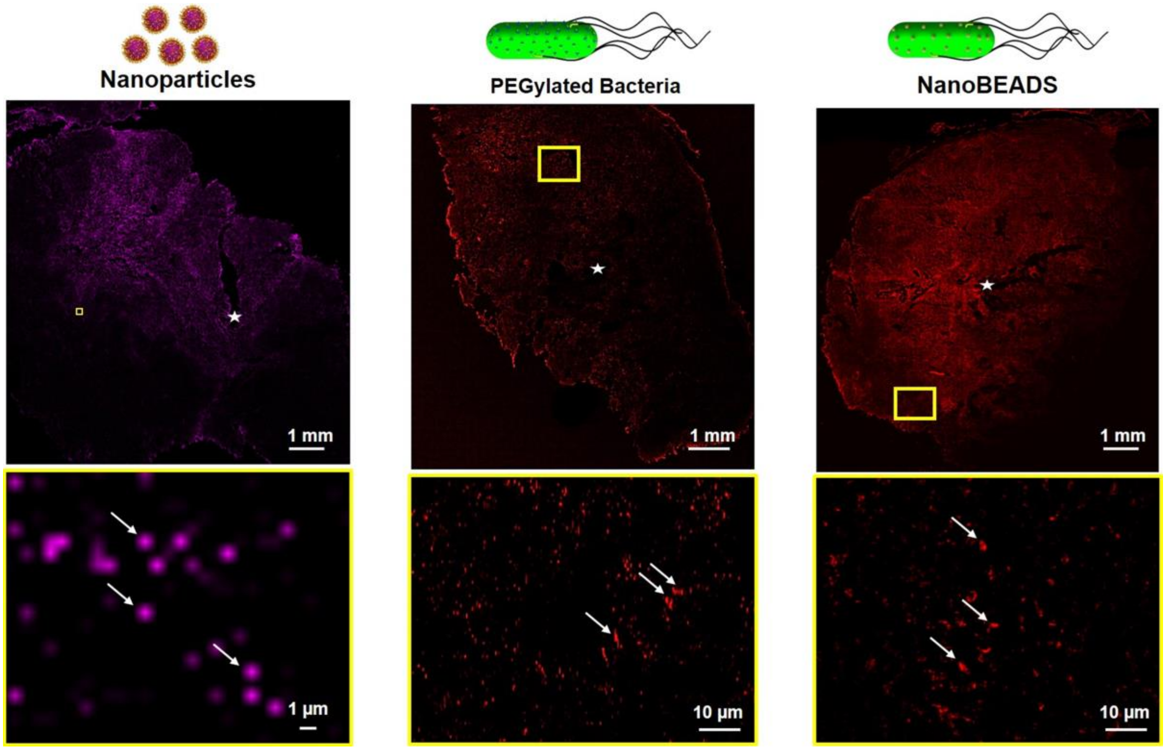

Figure S12. Intratumoral Transport of Therapeutic Agents in a Murine Breast Cancer Model. Confocal microscopy images showing the penetration of PLGA nanoparticles, PEGylated *S.* Typhimurium VNP20009, and NanoBEADS through 4T1 mammary tumors in BALB/c mice 48 h after intratumoral injection. The point of injection was considered to be at the end of the needle track, marked with asterisks on the images (top row). Zoomed-in micrographs with white arrows pointing to representative therapeutic agents (bottom row).

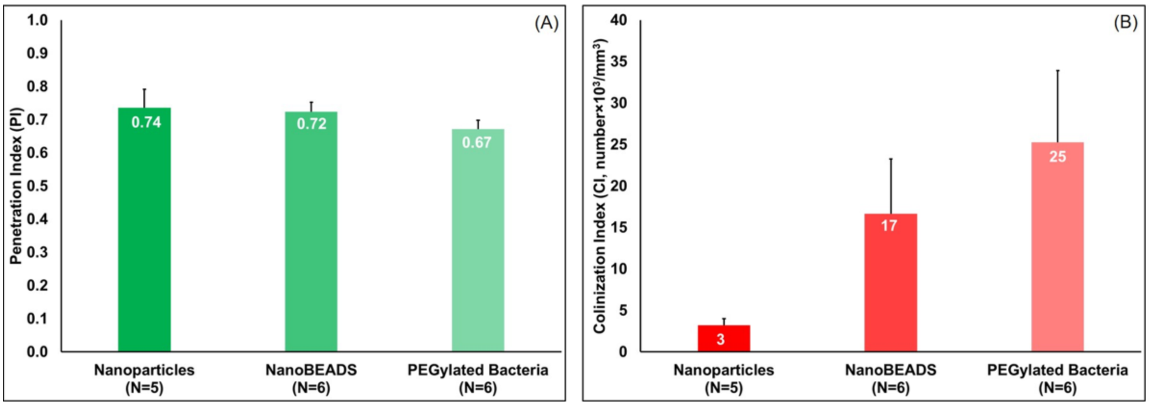

Figure S13. Quantitation of the Intratumoral Transport of Therapeutic Agents in a Murine Breast Cancer Model. Penetration Index **(A)** and Colonization Index **(B)** for PLGA nanoparticles, PEGylated *S.* Typhimurium VNP20009, and NanoBEADS through 4T1 mammary tumors in BALB/c mice 48 h after intratumoral injection. The point of injection is considered as the radial coordinate origin in PI calculations. Each NanoBEADS agent carries an average of 22 nanoparticles, thus, it significantly enhances the intratumoral retention of nanoparticles.

Table S1. Complete growth media for the cancer cell lines used in this study

Cell line	ATCC Catalog #	Cell Type	Complete Growth Media	Doubling Time [Hours]
HCT-116	CCL-247	Colon Carcinoma	McCoy's 5a + 10% (v/v) FBS	21
U87MG	HTB-14	Glioblastoma	EMEM + 10% (v/v) FBS	34
4T1	CRL-2539	Mammary Carcinoma	RPMI-1640 + 10% (v/v) FBS	23

II. Image Processing for Quantitation of the Spatial Distribution of Therapeutic Agents in Tumor Slices

We developed a custom image processing routine to quantitate and produce a 3D map of the therapeutic agents in tumor slices that were imaged using a laser scanning confocal microscope. Our algorithm is comprised of three components: *(i) Calibration of the size and intensity of the object of interest:* sample images containing sparsely distributed singular therapeutic agents were processed to define average object size or the number of pixels that represent a therapeutic agent. Next, an analytical function was fit to the distribution of fluorescence intensity along the *z*-axis for a series of representative *z*-stacked images, and fluorescent signal dissipation with respect to distance in the *z*-dimension was determined. Together, this data provided the object size information needed to accurately quantify the number of objects; *(ii) Conversion of the grayscale fluorescent intensity image into a binary image:* User-defined thresholding parameters including global and local gray-level intensity values were applied to convert grayscale input images into binary images; *(iii) Construction of the 3D distribution map:* using binary images produced in (ii) and the calibration data from (i), a compensation algorithm was utilized to enable the enumeration of fluorescent therapeutic agents in 40 µm-thick tumor slices.

II. A. Error Analysis

In order to determine the accuracy of our image processing routine, we compared the number of fluorescent objects detected by the image processing routine with the number of objects detected by manual counting. The error was calculated according to:

$$\%\ \text{Error} = \frac{|\mathrm{N}_{\mathrm{manual}} - \mathrm{N}_{\mathrm{automated}}|}{\mathrm{N}_{\mathrm{manual}}} \times 100,$$

where $\mathrm{N}_{\mathrm{manual}}$ is the number of objects determined by manual counting and $\mathrm{N}_{\mathrm{automated}}$ is the number of objects reported by the image processing routine. In the case of nanoparticles, the manual counting was carried out using scanning electron microscopy (SEM) images, whereas, for the larger bacteria and NanoBEADS, the manual counting was done from the confocal microscopy images. It should be noted that, given the co-localization of the nanoparticles and bacteria in the case of NanoBEADS (**Figure S6**), only the signal from bacteria (visualized at 543 nm excitation wavelength with a 553-624 band-pass filter) was

used for image processing to avoid any error due to variation in the number of PLGA nanoparticles (visualized at 633 nm excitation wavelength with a 635-750 band-pass filter). The bacterial nanoparticle load information was separately determined through analysis of SEM images (**Figure 1**). As shown in **Figure S14**, we found that the mean error in the bacteria and NanoBEADS case was 7.8 ± 1.9 % (n = 9). The error largely stemmed from the variability in bacteria orientation which leads to variation in their projected area. In the case of nanoparticles, the mean error was 13.1 ± 1.9 % (n = 22). The larger error was due to the smaller size of the nanoparticles combined with the variability in the 3D clustering of the

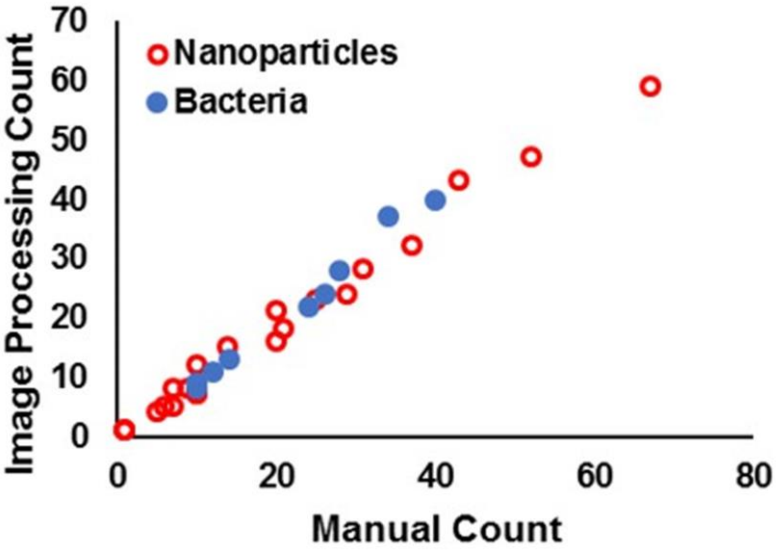

Figure S14. Image Processing Error Analysis. The error in quantitation of the spatial distribution of therapeutic agents was determined by comparing the number of fluorescent objects reported by our image processing routine with the number of fluorescent agents determined through manual counting. The mean error was found to be 7.8 ± 1.9 % in the case of bacteria and NanoBEADS and 13.1 ± 1.9 % in the case of nanoparticles.

nanoparticles.
